# Micro-magnetic resonance imaging study of live quail embryos during embryonic development

**DOI:** 10.1016/j.mri.2010.08.004

**Published:** 2011-01

**Authors:** Suzanne Duce, Fiona Morrison, Monique Welten, Glenn Baggott, Cheryll Tickle

**Affiliations:** a Division of Biological Chemistry and Drug Discovery, College of Life Sciences, University of Dundee, DD1 5EH Dundee, UK; b Division of Cell and Developmental Biology, College of Life Sciences, University of Dundee, DD1 5EH Dundee, UK; c Department of Biology and Biochemistry, University of Bath, BA2 7AY Bath, UK; d Department of Biological Sciences, Birkbeck College, University of London, London WC1E 7HX, UK

**Keywords:** Micro-MRI (μMRI), Safety, Quail, Embryo, Egg, In ovo, Embryonic development

## Abstract

Eggs containing live Japanese quail embryos were imaged using micro-magnetic resonance imaging (μMRI) at 24-h intervals from Day 0 to 8, the period during which the main body axis is being laid down and organogenesis is taking place. Considerable detail of non-embryonic structures such as the latebra was revealed at early stages but the embryo could only be visualized around Day 3. Three-dimensional (3D) changes in embryo length and volume were quantified and also changes in volume in the extra- and non-embryonic components. The embryo increased in length by 43% and nearly trebled in volume between Day 4 and Day 5. Although the amount of yolk remained fairly constant over the first 5 days, the amount of albumen decreases significantly and was replaced by extra-embryonic fluid (EEF). ^1^H longitudinal (T_1_) and transverse (T_2_) relaxation times of different regions within the eggs were determined over the first 6 days of development. The T_2_ measurements mirrored the changes in image intensity observed, which can be related to the aqueous protein concentrations. In addition, a comparison of the development of Day 0 to 3 quail embryos exposed to radiofrequency (rf) pulses, 7 T static magnetic fields and magnetic field gradients for an average of 7 h with the development of control embryos did not reveal any gross changes, thus confirming that μMRI is a suitable tool for following the development of live avian embryos over time from the earliest stages.

## 1 Introduction

Avian embryos are important experimental models for investigating embryonic development and in particular the processes that control the laying down of the body plan and organogenesis [1,2]. Their importance is due, at least in part, to the fact that they are encased within an egg which provides nearly all the components necessary for development. Most research on avian embryos investigates the development of the embryo [3], while the extra-embryonic and the non-embryonic components within the egg have attracted less attention [4,5], even though they are essential for embryonic development. The extra-embryonic components (e.g., yolk sac, allantois and amnion) are temporary structures participating in fundamental metabolic processes such as respiration, nutrition and excretion. The non-embryonic components of the egg (e.g., yolk, albumen and shell) provide nutrients and also physical and microbial protection for the growing embryo [4].

Micro-magnetic resonance imaging (μMRI) is a good method for investigating changes in the three-dimensional (3D) internal anatomy of optically opaque objects [6]. The MR images of fixed avian embryos [7–10] contain excellent anatomical detail and an MRI atlas of quail development has been produced [9]. Since MRI is a noninvasive and nondestructive technique, it is also ideally suited for visualizing live embryos in ovo. In ovo MRI images [11–14] allowed the visualization of yolk, albumen and embryo. Magnetic resonance imaging of live avian embryos in ovo is technically more demanding than imaging of fixed embryos, because of the movements of the live embryos. In addition, the increase in the size of the radiofrequency (rf) resonators needed to accommodate the whole egg results in a decrease in the signal-to-noise, and often in a reduction in spatial resolution. Ways to overcome these problems are to cool the eggs prior to imaging as it reduces embryonic movement and also to use fast image acquisition experiments. Recently, longitudinal in ovo studies of chick [15] and quail [16] have been reported that study embryonic development over time. Bain et al. [15] studied embryonic chick development from Day 12 through to hatching; Hogers et al. [16] presented quail images at 48-h intervals from Day 3 to Day 11 to investigate the development of the embryonic heart.

In this article, we present images of quail eggs obtained at 24-h intervals from Day 0 to Day 8 to follow the embryonic development and quantify volumetric changes in the embryo and also in the extra- and non-embryonic components. Volumetric measurements were made and temporal changes quantified in this longitudinal study. In order to interpret the changes in image contrast observed during development, the ^1^H longitudinal (T_1_) and transverse (T_2_) relaxation times of the various extra- and non-embryonic materials within quail eggs at different stages of development were determined by MRI. An ex vitro NMR proton relaxation study of unfertilized hen's albumen and yolk has demonstrated that changes in transverse relaxation in the albumen correlated with increased protein concentrations and can be related to egg quality [17].

The usefulness of μMRI to follow quail embryonic development over time relies on embryonic development proceeding normally, but there have been concerns that the strong magnetic fields and magnetic field gradients associated with MRI could affect development. No adverse effects on chick embryo development have been observed at low magnetic fields of 1.5 T [18–20] nor on survivability and hatching when in ovo chick embryos from Day 12 onwards were exposed to moderate cooling and high static 7 T magnetic fields [15]. However, the effects of high magnetic fields on early avian development have not been assessed. Therefore we exposed in ovo quail embryos from Day 0 to Day 3 to high static 7 T magnetic fields, linear magnetic field gradients and 300 MHz rf pulses. Embryos were fixed at Day 7 and compared with embryos from control eggs that had been removed from the incubator for the same period of time but not subjected to magnetic fields, as well as with embryos from eggs left in the incubator until Day 7.

## 2 Methods

### 2.1 Quail egg handling

Fertilized Japanese quail (*Coturnix japonica*) eggs were obtained from Rosedean Quail (Huntingdon, Cambridgeshire, UK). The day the eggs arrived was designated as Day 0. The eggs were imaged vertically, with air sac uppermost, in a plastic egg holder inside the rf resonator. After imaging, the eggs were placed in the same vertical orientation in humidified VWR incubators (VWR International, Ltd., Lutterworth, Leicestershire, UK) at 38°C. Each day, the eggs were removed from the incubator, cooled for 3 min in running tap water and dried before imaging. Cooling the eggs prior to imaging has been shown to reduce embryonic movements that degrade image quality [15]. After imaging, the eggs were immediately returned to the incubator.

### 2.2 Micro-MRI Instrumentation

Micro-MRI data were acquired on a Bruker Avance FT NMR spectrometer with a wide bore 7.1 T superconducting magnet resonating at 300.15 MHz for ^1^H. A birdcage rf resonator with an internal diameter of 30 mm was used. The rf resonator was tuned and the magnet shimmed for each sample. All acquisitions were made at 19°C. The field of view was 32 mm and in-plane spatial resolution was 0.25 mm/pixel. Two acquisition sequences were collected and averaged to improve the signal-to-noise ratio and reduce artifacts [21]. A 128×128×128 rapid acquisition relaxation enhanced (RARE) pulse sequence was used with RARE factor of 8. Recycle time (T_R_) of 500 ms and an effective echo time (T_E_) of either 20 or 30 ms were used. The MRI data took less than 35 min to acquire.

Relaxation measurements were determined from two-dimensional 128×128 data sets from a sagittal plane through the eggs with field of view of 30 mm and slice thickness of 1 mm. Recycle time was 15 s to avoid saturation of the magnetization. An inversion recovery (180°-TI-90°) imaging pulse sequence was used to measure the T_1_ relaxation times: eight inversion times (TI) that ranged from 0.5 to 15 s were applied. Echo time was 4 ms. A Carr-Purcell-Meiboom-Gill spin-echo imaging pulse sequence was used to measure T_2_ relaxation times [21]. A train of 16 echoes was acquired and the delay (*τ*) between 180° pulses was 10 ms. Single exponential relaxation times were calculated from experimental data using Bruker Paravision software.

### 2.3 MRI data analysis

Fourier-transformed, 3D MRI data were visualized using Amira imaging PC-based software (Visage Imaging, Inc., San Diego, CA, USA). This allowed 2D slices to be viewed from any angle within the 3D data set and regions of interest segmented, finite element meshes were generated and then surface rendered. Thus anatomy could be visualized and volumetric measurements determined.

### 2.4 MRI biosafety experiments

Quail eggs between Incubation Day 0 and 3 were exposed to a high static 7 T magnetic field, linear magnetic field gradients (with maximum gradient amplitude of 200 mT/m) and 300 MHz rf pulses for several hours (average of 7 h) (test group). This long exposure time was to determine whether the high magnetic fields had any adverse affects upon embryonic development. Eggs removed from the incubator for the same period of time but not subjected to external magnetic fields made up the control group. After MRI scanning, test and control eggs were returned to the incubator until Day 7. A third group of eggs (incubator group) remained continuously in the incubator until Day 7. At Day 7, the quail embryos were removed from the three groups of eggs, fixed in 4% paraformaldehyde in 0.1 M phosphate-buffered saline (PBS) and left overnight at 4°C. The specimens were then washed with PBS. These embryos were observed under a microscope to assess and record the developmental stage using Hamburger/Hamilton staging [22] to monitor whether development was normal.

## 3 Results and discussion

### 3.1 Longitudinal μMRI study of quail eggs

**Fig. 1:**
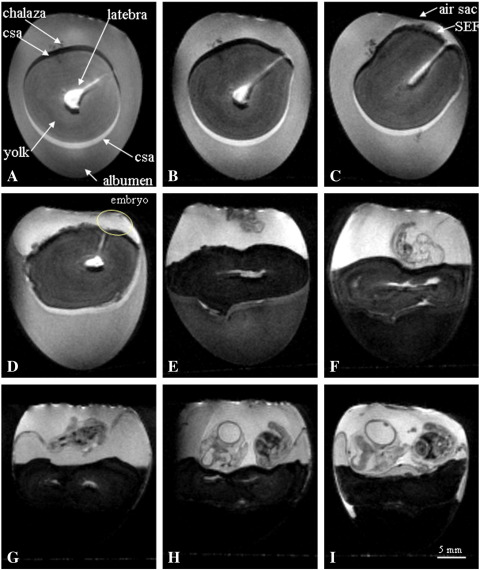
Two-dimensional RARE-8 vertical MR images from a 3D data set of a quail egg acquired longitudinally during the first 8 days of incubation. (A) Day 0; (B) Day 1; (C) Day 2; (D) Day 3; (E) Day 4; (F) Day 5; (G) Day 6; (H) Day 7; (I) Day 8. csa=Chemical shift artifact.

**Fig. 2:**
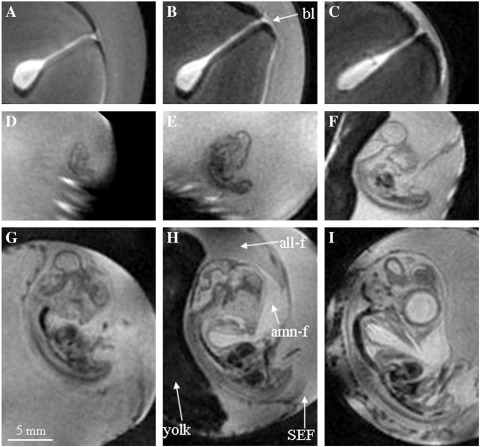
Two-dimensional RARE-8 MR images of the sagittal plane of a developing quail embryo in ovo acquired longitudinally during the first 8 days of incubation. (A) Day 0; (B) Day 1; (C) Day 2; (D) Day 3; (E) Day 4; (F) Day 5; (G) Day 6; (H) Day 7; (I) Day 8. bl=Location of the blastoderm; all-f=allantoic fluid; amn-f=amniotic fluid.

**Fig. 3:**
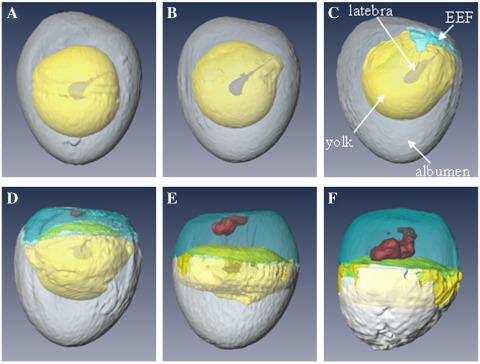
Three-dimensional surface-rendered RARE-8 MR images of a vertical view of a quail egg acquired longitudinally during the first 5 days of incubation: (A) Day 0; (B) Day 1; (C) Day 2; (D) Day 3; (E) Day 4; (F) Day 5. Yolk (yellow), albumen (white), EEF (turquoise), embryo (dark red) and latebra (purple) are produced by digital segmentation using Amira software.

The main aim of the study was to undertake longitudinal μMRI studies of quail embryos developing within their eggs and then quantify the developmental changes in the embryos and the extra- and non-embryonic regions. Six eggs were studied over an 8-day period. On the day the eggs arrived (Day 0), they were imaged using 3D RARE-8 MRI sequence. This fast spin-echo imaging sequence takes about 35 min to obtain, after which the eggs were placed in the incubator. Consecutive 3D images were acquired at 24-h periods. Representative MRI images are shown in Figs. 1, 2 and 3; all these images are from the same egg. Images with equivalent letters were acquired at the same time points and originate from the same MRI data set. Fig. 1 displays a 2D vertical slice from the whole egg; Fig. 2 shows 2D images of the sagittal plane through the developing quail embryo; and Fig. 3 is a 3D surface rendering of various components after segmentation using Amira software. The yolk is represented in yellow, albumen in white, extra-embryonic fluid (EEF) in turquoise, embryo in dark red and latebra in purple.

The yolk, albumen, chalaza, latebra and the neck of the latebra are all visible in the Day 0 image (Fig. 1A). The yolk is spherical and lies in the center of the egg surrounded by albumen. The latebra is in the center of the yolk and the neck of the latebra emanates outwards towards the blastoderm. The bright crescent visible at the bottom and dark crescent at the top of the yolk are chemical shift artifacts that arise because the chemical shift of water protons is about 3.5 parts per million higher than that of the lipid protons; so the water and lipid images are slightly displaced with respect to each other along the axis in which the “read” magnetic field gradient is applied.

The MR images acquired at 24-h intervals give very informative 3D snapshots of the changes that are occurring in the structure of the egg during embryonic development. At Day 1, the spherical shape of the yolk becomes slightly distorted in the region around the blastoderm (Fig. 1B). By Day 2, there are significant changes in the shape of the upper surface of the yolk (Fig. 1C), and the yolk has moved so that it is nearly touching the air sac. The air sac is not visible by MRI and is located at the top of the egg above the concave upper albumen surface (Fig. 1C). There does not seem to be much change in the shape of the lower region of the Day 2 yolk (Figs. 1C and 3C). In contrast, the shape of the yolk in the region near the blastoderm has become distorted and protrudes upwards. The blastoderm is attached to the vitelline membrane; gradually the membrane ruptures allowing the expansion of the yolk sac as it fills with sub-embryonic fluid (SEF). By Day 3, the differences in the shape of the uneven upper surface of the yolk and its lower curved surface are very distinct (Fig. 1D). By Day 4, the vitelline membrane has completely ruptured, while the yolk sac membrane remains encompassing the yolk and SEF. Figs. 1E and 3E show the yolk distributed across the middle of the egg. The yolk is separating two aqueous regions: above the yolk is the aqueous EEF with high image intensity and below is the lower image intensity albumen. This arrangement continues for the subsequent 3 days (Days 5 to 7 in Fig. 1F–H).

Fig. 1A–D shows how the orientation of the neck of the latebra changes with time. At Day 0, the neck of the latebra lies at about 60° from vertical axis extending from the latebra to the surface of the yolk near the blastoderm. By Day 3, the neck of the latebra is about 20° from the vertical axis. It is known that the blastoderm will move to the uppermost surface of the yolk [23]. The eggs were stored on their side during transit but on arrival the eggs were incubated vertically with air sac uppermost, an arrangement chosen because the bore of the magnet was too narrow to image the eggs on their side. As a result of changing the orientation of the egg, the blastoderm migrates upwards over the yolk surface and the neck of the latebra moves with it. At Day 4, the shape of the latebra in the center of the yolk changes significantly from a spherical region to a flat horizontal star-like structure (Fig. 1E). The latebra becomes smaller and less distinct after Day 6 (Fig. 1G).

**Fig. 4:**
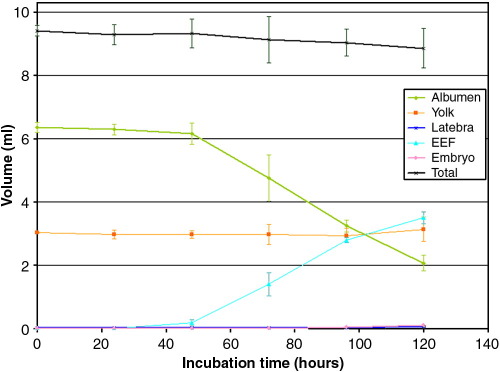
Changes in the volume (ml) of albumen (green), yolk (orange), EEF (light blue), latebra (dark blue), embryo (dark red) and total fluid content (black) of quail eggs (*n*=3) plotted against incubation time (hours). Standard deviation used for error bars. The volumes are tabulated as supplementary data.

Segmentation and 3D surface rendering of the embryo, yolk, albumen, EEFs and latebra during first 120 h of development were carried out for three eggs in the longitudinal study. This allowed the 3D changes in the shape, location and volume of the various components during embryonic development to be visualized (Fig. 3) and quantified (Fig. 4) (Supplementary Data Table S1). At Day 0 (Fig. 3A), about 70% of the egg is albumen and the rest is yolk. Over a 120-h period, the mean total volume of fluids in the egg decreases by 5.8% from 9.41 to 8.86 ml. This is due to water loss by evaporation through the shell, even though the incubators are humidified to help reduce water loss. The volume of yolk in the quail egg is about 3 ml and this does not significantly change during early embryonic development. In contrast, dramatic changes in the aqueous regions are detected after 48 h of incubation. An aqueous region becomes visible above the yolk, which includes SEF (Fig. 1C). From Day 0 to Day 5, the volume of albumen decreases by 67.6% from 6.37 to 2.06 ml. Much of the reduction in the volume of albumen is due to movement of water to SEFs and the other to EEFs; by Day 5, the volume of EEF has increased to 3.51 ml. The EEFs consist of fluid lying under the embryo in the sub-germinal space and fluids within extra-embryonic cavities enclosed by the chorion and amnion. The SEF is less dense than the albumen [24] and lies within the yolk sac above the yolk. The amniotic fluid around the embryo and the fluid in the allantois can be distinguished in high-resolution images of the embryo (annotated in Fig. 2H). By Day 7, the image intensity of the allantois fluid is lower (darker image) than that of the amniotic fluid. The drop in image intensity in allantois arises from an increase in biomolecules including paramagnetic iron which decrease the water's transverse relaxation rate.

**Fig. 5:**
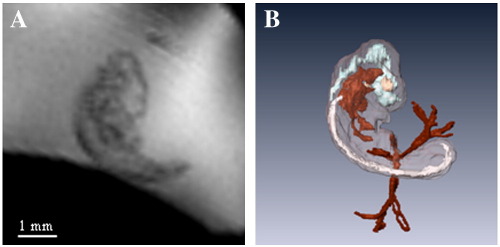
MR images from 3D data set of quail embryo in ovo at Day 3 incubation. (A) Two-dimensional sagittal MR image of the developing quail embryo; (B) 3D surface rendering of embryo (semi-transparent pink), vessels (red), spine (white), brain (light blue) and eyes (cream) after digital segmentation using Amira software.

The embryo first becomes visible in the MR images of quail eggs on Day 3 (Fig. 1D). The sagittal crown-rump length of the Day 3 embryo is around 4 mm (Fig. 5A). Some anatomical structures can be made out and the image digitally segmented to produce 3D representations (Fig. 5B) of features of the vessels (red), spine (white), brain (light blue) and eyes (cream). Sagittal crown-rump length increases to 7 mm (Day 4), 10 mm (Day 5) and 14 mm (Day 6), respectively (Fig. 2). The volume of the embryo at Day 3 is about 0.02 ml which increases to 0.038 ml at Day 4 and 0.105 ml at Day 5; thus the volume of the embryo nearly trebles between Day 4 and Day 5. The Day 3 embryo lies close to the top of the egg as the embryo is slightly less dense compared to the other aqueous fluids (Fig. 1D). By Day 5, the embryo lies relatively lower, a few millimeters above the yolk, head downwards. At Day 7, the embryo is lying on its side above the yolk's upper surface and the amniotic fluid is visible (Fig. 2H). These positional changes reflect changes in the embryo's density relative to the density of the EEF and the yolk [4,24]. At Day 3, when the vasculature is still forming, it would be advantageous for the embryo to be near the shell to ensure adequate oxygen availability. As the embryo grows and its blood supply matures, it would benefit from the extra physical protection provided by being nearer the center of the egg.

### 3.2 MRI relaxation study of quail eggs

The image contrast between different components within the egg changes noticeably during embryonic development. MRI relaxation measurements permit the relaxation times of different regions within the egg to be investigated. The longitudinal (T_1_) and transverse (T_2_) ^1^H relaxation times of the albumen, yolk, EEF and latebra within the quail eggs were determined during early stages of development and tabulated (Table 1). The T_1_ and T_2_ relaxation times of the yolk ranged between 0.34 and 0.42 s and between 24 and 31 ms, respectively, and did not change significantly during development. Egg yolk is an exceedingly complex, microheterogeneous substance [25], and optical microscopy reveals yolk spheres, granules and lipoprotein complexes suspended in an aqueous solution called yolk plasma. The yolk's insensitivity to ^1^H relaxation times suggests that its microstructure is quite stable during early development.

By Day 2, both the T_1_ and T_2_ relaxation times of the EEF are significantly longer than that of the albumen. Hence this EEF has a higher signal intensity (appears brighter) compared to the albumen in the T_2_-weighted RARE images (Fig. 1C). At Day 3, the T_2_ relaxation time in EEF and albumen is 197 and 74 ms, respectively. The T_2_ relaxation time of water in albumen drops significantly from Day 3 onwards so that by Day 6 its relaxation time was below 20 ms. This drop results in the decrease in image signal intensity arising from the albumen region over time and explains why the albumen in these RARE images appears black by Day 6 (Fig. 1G). Laghi et al. [17] demonstrated in an ex vitro quantitative NMR proton relaxation study of unfertilized hen's albumen and yolk that there is a direct relationship between the transverse (1/T_2_) relaxation rate of the albumen and protein concentration. Ovalbumin proteins contain exchangeable protons with very short T_2_ relaxation times, and the exchange between these protons and water protons reduces the observed water T_2_ relaxation times in a predictable manner. It is known that the albumen contains a range of different proteins and that their concentration increases significantly during embryonic development [4,24]. Thus the major decrease in both the T_1_ and T_2_ relaxation times of albumen can be linked to the increase in protein concentration. Thus, the longer observed relaxation times of the EEF are consistent with the much lower concentration of proteins in this fluid [24].

### 3.3 MRI biosafety study

The usefulness of MRI to monitor the development in vivo will be reduced if MRI scanning leads to delayed development or to developmental defects. Therefore the effects of rf pulses, high static magnetic fields and varying magnetic gradients on the first 3 days of quail embryonic development were investigated. Quail eggs were removed from the incubator during the first 3 days of development and exposed for an average of 7 h to high static 7 T magnetic field, linear magnetic field gradients (with maximum gradient amplitude of 200 mT/m) and 300 MHz rf pulses (test group). These exposures were longer than those typically used to capture images but were chosen in order to test the biosafety of MRI. Control group eggs were removed from the incubator for the same period of time on each day but not subjected to an external MRI magnetic field (control group). Test and control eggs were then returned to the incubator until Day 7. In addition, a third group of eggs were incubated continuously until Day 7 (incubator group). After which all the embryos were removed, fixed and their development assessed.

The results are shown in Table 2. The median embryonic stage of the test and control groups was 34, while that of the incubator group was 35. The Kolmogorov–Smirnov (KS) test was used to estimate the probability of whether the distribution of embryo stages in the test group is different from that of the control group. Their distributions were very similar with a *P* value of nearly 1.0 and a KS distance (*D*) of only 0.031 (Supplementary Data Figure S1), which indicates that their profiles were almost identical. In contrast, using the KS test to compare the embryo stages in the control and incubator groups produced a very low *P* value of .003 with a larger KS distance (*D*) of 0.502. The slight delay in development in both the test and control groups compared with the incubator group is expected because the temperature of the egg drops from 38°C to 19°C on removal from the incubator and this is known to slow down embryonic development [4]. The % of embryos in each group with retarded development (i.e., had not reached Stage 33 by the end of the experiment) and/or with developmental defects is also shown in Table 2. The developmental defects, which were seen in all three groups, included misshapen embryos and absence of eyes. There is only a small difference in the % of these abnormal embryos in the three groups: 13% in the control and incubator groups and 15% in the test group. Taken altogether, all these results show that high external magnetic fields, magnetic field gradients and rf pulse had no apparent adverse effect upon the early development of quail embryos.

## 4 Conclusions

Micro-MRI can be safely used to follow normal development of live quail embryos, in ovo, over the first 8 days of development. At the earliest stages, non-embryonic structures such as the latebra are revealed very clearly but few details can be made out in the embryo until 3 days of development. It may be possible to improve visualization of the early embryo by injecting doses of contrast agents into the egg that do not harm the embryo. At later stages, we have shown that MRI can be used noninvasively to measure the growth of the embryo in terms of both crown-rump length and volume. It is possible to measure growth of particular organs within the embryo [16]. Thus MRI could be useful for monitoring gross effects of exogenous agents injected into the egg on embryonic development over time. We have also shown that MRI reveals differences between albumen and other fluids in the egg and can even distinguish between amniotic and allantoic fluid. The temporal changes in the ^1^H longitudinal (T_1_) and transverse (T_2_) relaxation times of aqueous components within quail eggs are linked with changes in the concentration of soluble proteins and carbohydrates [17]. Finally, the imaging of the embryo developing within the intact egg gives a rare insight into the physical relationship between it and the other components in the egg.

## Figures and Tables

**Table 1:** MRI ^1^H longitudinal (T_1_) relaxation times (s) and transverse (T_2_) ^1^H relaxation times (ms) of the various components of quail eggs acquired during the first 6 days of incubation

	Day 0	Day 1	Day 2	Day 3	Day 4	Day5	Day 6
*T1 (s)*							
Albumen	2.2±0.2	2.05±0.02	2±.01	1.82±.17	1.51±.06	1.36±0.07	1.12±.08
Yolk	0.34±.02	0.35±.02	0.38±.02	0.37±.001	0.32±.05	0.42±.02	0.42±.02
Embryonic fluid			2.52±.2	2.8±.2	2.75±.27	2.59±.12	2.7±.13

*T2 (ms)*							
Albumen	101±5	98±6	99±7	74±13	49±9	26±2	19±3
Yolk	24±2	28±1	28±1	29±2	31±6	31±2	30±2
Latebra	50±6	60±9	51±3	51±5	54±10	60±10	55±9
Embryonic fluid			130±12	197±32	201±73	180±20	183±25

The data is an average of four T_1_ data sets and five T_2_ data sets and standard deviation given.

**Table 2:** Results of MRI biosafety study

No. of embryos	Stage 36	Stage 35	Stage 34	Stage 33	Below Stage 33	Small or deformed embryos	Median	Skew
Control	1	12	11	3	2	13%	34	−0.71
Magnet	2	12	12	3	3	15%	34	−0.59
Incubator	6	13	1	0	0	13%	35	0.113

Number of embryos at each developmental stage was recorded for the control, test and incubator groups; plus % of embryos that had developmental defects and/or retarded development was calculated. The median stage and skew are tabulated.
