# Supplementary material for: Micro-magnetic resonance imaging study of live quail embryos during embryonic development
Source: Magn Reson Imaging. 2011 Jan;29(1-6):132–9. doi: 10.1016/j.mri.2010.08.004 (PMC3006493; doi:10.1016/j.mri.2010.08.004)

**Supplementary Data**

**Supplementary Data Table S1:** The volume (ml) of albumen, yolk, latebra, extra-embryonic fluid (E-EF), embryo and total fluid within quail eggs at consecutive incubation days. The data represent an average of three data sets at each time point.

| **Vol (ml)** | **Day 0** | **Day 1** | **Day 2** | **Day 3** | **Day 4** | **Day 5** |
| --- | --- | --- | --- | --- | --- | --- |
| **Albumen** | 6.37±0.16 | 6.29±0.17 | 6.17±0.34 | 4.75±0.73 | 3.24±0.18 | 2.06±0.38 |
| **Yolk** | 3.03±0.01 | 2.97±0.14 | 2.97±0.012 | 2.96±0.31 | 2.94±0.23 | 3.14±0.37 |
| **Latebra** | 0.02±0.002 | 0.02±0.002 | 0.02±0.003 | 0.02±0.003 | 0.03±0.009 | 0.05±0.02 |
| **E-EF** | 0.00 | 0.00 | 0.18±0.1 | 1.40±0.37 | 2.80±0.08 | 3.51±0.19 |
| **Embryo** | 0.00 | 0.00 | 0.00 | 0.02±0.01 | 0.038±0.01 | 0.105±0.02 |
| **Total** | 9.41±0.17 | 9.29±0.31 | 9.33±0.45 | 9.14±1.1 | 9.04±0.51 | 8.86±0.81 |

**Supplementary Data Figure S1:** Kolmogorov-Smirnov cumulative fraction graph showing the distribution of embryonic stages of the Day 7 embryos from quail eggs in the control, test and incubator groups. The D value for control-test and control-incubation data was 0.031 and 0.502 respectively.


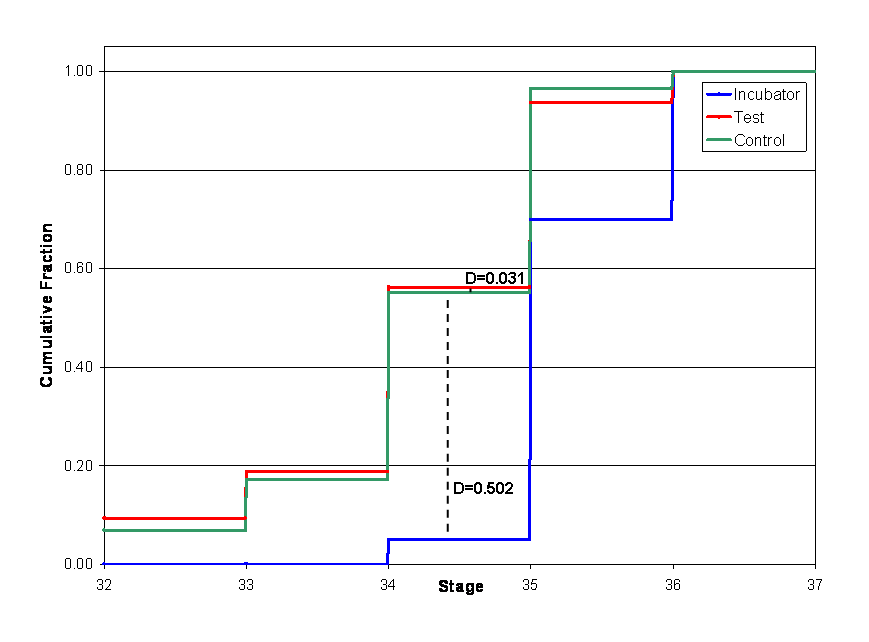

Supplement: Supplementary file 1 — Supplementary Data. [file mmc1.doc]
